# Standards in Pupillography

**DOI:** 10.3389/fneur.2019.00129

**Published:** 2019-02-22

**Authors:** Carina Kelbsch, Torsten Strasser, Yanjun Chen, Beatrix Feigl, Paul D. Gamlin, Randy Kardon, Tobias Peters, Kathryn A. Roecklein, Stuart R. Steinhauer, Elemer Szabadi, Andrew J. Zele, Helmut Wilhelm, Barbara J. Wilhelm

**Affiliations:** ^1^Pupil Research Group, Centre for Ophthalmology, University Hospitals Tübingen, Tübingen, Germany; ^2^Department of Ophthalmology and Visual Sciences, University of Wisconsin School of Medicine and Public Health, Madison, AL, United States; ^3^Institute of Health and Biomedical Innovation, Queensland University of Technology, Brisbane, QLD, Australia; ^4^School of Biomedical Sciences, Queensland University of Technology, Brisbane, QLD, Australia; ^5^Queensland Eye Institute, Brisbane, QLD, Australia; ^6^Department of Ophthalmology and Visual Sciences, University of Alabama at Birmingham, Birmingham, AL, United States; ^7^Neuro-Ophthalmology Division, University of Iowa and Iowa City VA Healthcare System, Iowa City, LA, United States; ^8^Department of Psychology, University of Pittsburgh, Pittsburgh, PA, United States; ^9^VA Pittsburgh Healthcare System, VISN 4 MIRECC, University Drive C, Pittsburgh, PA, United States; ^10^Department of Psychiatry, University of Pittsburgh School of Medicine, Pittsburgh, PA, United States; ^11^Developmental Psychiatry, University of Nottingham, Nottingham, United Kingdom; ^12^School of Optometry and Vision Science, Queensland University of Technology, Brisbane, QLD, Australia

**Keywords:** clinical standards, pupillography, application of pupillography, stimulus characteristics, parameters of evaluation, analysis, pupillometry

## Abstract

The number of research groups studying the pupil is increasing, as is the number of publications. Consequently, new standards in pupillography are needed to formalize the methodology including recording conditions, stimulus characteristics, as well as suitable parameters of evaluation. Since the description of intrinsically photosensitive retinal ganglion cells (ipRGCs) there has been an increased interest and broader application of pupillography in ophthalmology as well as other fields including psychology and chronobiology. Color pupillography plays an important role not only in research but also in clinical observational and therapy studies like gene therapy of hereditary retinal degenerations and psychopathology. Stimuli can vary in size, brightness, duration, and wavelength. Stimulus paradigms determine whether rhodopsin-driven rod responses, opsin-driven cone responses, or melanopsin-driven ipRGC responses are primarily elicited. Background illumination, adaptation state, and instruction for the participants will furthermore influence the results. This standard recommends a minimum set of variables to be used for pupillography and specified in the publication methodologies. Initiated at the 32nd International Pupil Colloquium 2017 in Morges, Switzerland, the aim of this manuscript is to outline standards in pupillography based on current knowledge and experience of pupil experts in order to achieve greater comparability of pupillographic studies. Such standards will particularly facilitate the proper application of pupillography by researchers new to the field. First we describe general standards, followed by specific suggestions concerning the demands of different targets of pupil research: the afferent and efferent reflex arc, pharmacology, psychology, sleepiness-related research and animal studies.

## Introduction

Otto Lowenstein and Irene Loewenfeld established a new era of pupil research with the development of infrared-video-pupillography (1). In the first instance, each single picture of the pupil was analyzed manually, before Lowenstein and Loewenfeld introduced the first on-line analysis with their newly constructed photoelectric pupillograph in 1947. It was not until the late seventies, when videotaping became possible, allowing recording of the pupil diameter continuously in darkness via infrared-videography with a combined computerized data analysis. Based on the knowledge of Irene Loewenfeld's outstanding life work (2), and particularly since the description of melanopsin expressing intrinsically photosensitive retinal ganglion cells (ipRGCs), there has been an increased interest and broader application of pupillography in ophthalmology as well as other fields including psychology and psychiatry.

IpRGCs, a subclass of retinal ganglion cells, are capable of detecting light directly via the photopigment melanopsin (3, 4), in addition to receiving input from the traditional extrinsic pathway via photoreceptors of the outer retina. Thus, the pupillary light reflex consists of rhodopsin-driven rod responses, opsin-driven cone responses and melanopsin-driven ipRGC responses (5–11). Depending on the stimulus paradigms, such as stimulus size, brightness, duration, and wavelength as well as background illumination and adaptation state of the retina, pupillary responses reflect these different response components.

Color pupillography currently plays an important role in different clinical and research areas. On the one hand, there is fundamental basic science being performed based on pupillographic animal studies with knockout models (12–14), but also on the cellular level (15, 16). On the other hand, there are pupillographic clinical studies in humans in order to better understand the pupil circuitry [e.g., (17, 18)] and the pathomechanism and remaining retinal functionality of certain diseases, e.g., glaucoma (19–23), Retinitis pigmentosa (9, 24–27), age-related macular degeneration (28–30), diabetes (31–33) or hereditary optic neuropathy (34). Furthermore, pupillography comes into use in clinical observational and therapy studies like gene therapy of hereditary retinal degenerations (35), studies on attention-modulation (36), in chronobiology [(37–39), for a review see (40)] and in psychopathology, psychiatric disorders and neurodegenerative conditions (41–45). Additionally, pupillography is indispensable in sleepiness-related research and the pupillographic sleepiness test (PST) has been developed into an objective measures of day time sleepiness (46, 47). In the last years, automated pupillography also found its way into the evaluation of patients in intensive care units, particularly using pupillary abnormalities in the management of severe traumatic brain injury as an indicator for an increased intracranial pressure (48) or in the management of analgesia (49, 50).

In all disciplines, one takes advantage of analyzing the pupil behavior and pupillary responses to specific stimuli: Pupil measurements are contactless, easily accessible, and objective, with only minor cooperation required from the examined participant.

The number of researchers studying the pupil is increasing, as are the number of publications, which increased almost exponentially over the past 50 years. In order to achieve a higher comparability of pupillographic studies worldwide and to increase the scientific weight of pupillography and pupil research, standards in pupillography regarding methodology including recording conditions, stimulus characteristics, as well as an agreement about parameters of evaluation are needed. Standards particularly facilitate the steps to perform a technically appropriate pupillographic procedure and to analyze and report the data properly, and pupillographic guidelines serve as a common basis for pupillography in scientific and clinical applications between different labs.

Visual electrophysiology, which allows for an objective evaluation of the visual pathway similar to pupillography, was confronted with similar requirements: Research groups started to develop sophisticated stimulus paradigms, leveraged by advances of technology and companies started to implement them into electrophysiological equipment. With the growing and widespread importance of visual electrophysiology for research and clinical routine, it became apparent that a common agreement of the principles of conducting visual electrophysiological tests was necessary in order to guarantee the comparability of results obtained in different labs, especially in clinical settings. The International Society for Clinical Electrophysiology of Vision (ISCEV)^1^ recognized the need for standardization at its founding in 1961 (51, 52), but it took until 1989 until the first standard for electroretinography was published (53). These standards describe a set of basic stimuli that should be recorded in electrophysiological tests performed clinically. Marmor and Zrenner, two of the authors of the standards, state: “This ensures that electrophysiologic testing will always produce a core of data that is recognizable and comparable everywhere, whether for clinical or research purposes. This program of standardization has been highly successful. Today, most publications using visual electrophysiology refer to these standards and the major manufacturers of clinical electroretinographic equipment have incorporated them into their stimulus protocols.” (54). Nowadays, standards are available for the different examination techniques in visual electrophysiology (55–59) which are accompanied by guidelines for calibration of stimulus and recording parameters (60) as well as a general guide to visual electrodiagnostic procedures (61). These standards and documents could serve as blueprints for analogous standards concerning stimulus and recording parameters of pupillography.

Initiated at the 32nd International Pupil Colloquium 2017 in Morges, Switzerland, the aim of this manuscript is to outline standards in pupillography based on current knowledge and experience of pupil experts in order to achieve greater comparability of pupillographic studies. It is divided into two major parts with general recommendations and specific application areas of pupillography:

- Part: General standards for Pupillography
  Data collection and processing
  Reported/provided data
- Part: Specific standards for Pupillography
  1. The afferent pupillary pathway
    1.1 Rod and cone photoreceptor contribution to the pupil light reflex
    1.2 Melanopsin - The Post-Illumination Pupil Response (PIPR)
    1.3 Special clinical applications
  2. The efferent pupillary pathway
  3. Pharmacology
  4. Psychology and Psychiatry
  5. Sleepiness-Related Pupillary Oscillations
  6. Animals

The first part is concerned with general standards for pupillography which should be reported in any pupillographic study. It contains basic information about the pupillographic device, the adaptation status of the retina, the stimulus characteristic as well as general information about the examined species. The second part provides specific standards regarding the specific demands of different areas of pupil research: the afferent pupillary pathway, the efferent pupillary pathway, pharmacology, psychology and psychiatry, sleepiness-related research and animal studies. It begins with a description of appropriate stimulus characteristics, followed by a presentation of appropriate response analysis parameters.

## I. Part: General Standards for Pupillography

### Data Collection and Processing

In addition to time series data, all pupillographic recordings should include data based on the Minimum Information about a Neuroscience Investigation (MINI), published by the CARMEN consortium (62). These data allow for the interpretation and the evaluation of the data by independent readers and can facilitate later computational access and analysis. Table 1 gives an overview of these guidelines adapted for pupillography.

**Table 1:**
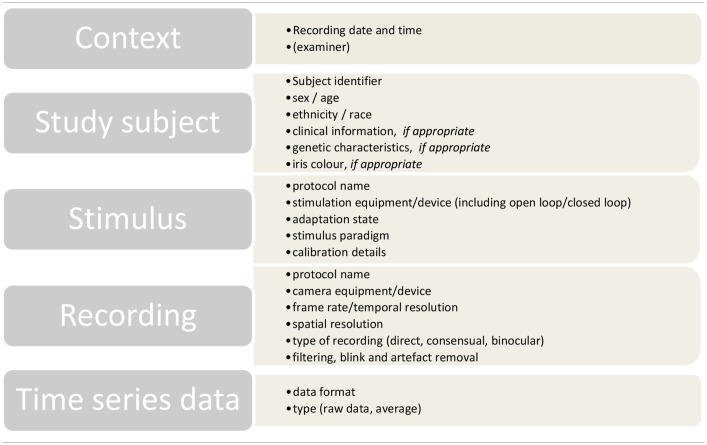
Overview of the MINI recommendations of the CARMEN consortium (62) adapted for pupillography.

### Reported/Provided Data

Information on the following topics is essential and recommended being addressed in any paper containing typical experiments performed with pupillography.

#### Pupillographic Device

The pupillographic device should be sufficiently explained to allow replication. That requires primarily whether a commercially available device (including name, city, and country of producer) or a self-built pupillograph has been used. The different components should be outlined together with the characteristics of the device including spatial and temporal resolution of the camera and the method of measurement (direct vs. consensual vs. binocular measurement of the pupils). Moreover, the method of stimulus presentation should be reported as stimuli might be either presented as a full-field (Ganzfeld bowl, mini-Ganzfeld bowl/tube, Maxwellian view/glasses) or focally on a hemisphere (perimetry) or a flat monitor (campimetry). The respective distance between the examined participant's cornea and the presented stimulus' location is required.

#### Demographic Data

Information on the examined species is crucial; this includes whether human subjects or animals were tested and should always be accompanied by a statement of keeping the conditions of ethical standards according to the Declaration of Helsinki and animal standards. The age range is likewise required as information of the sex and specific features like clinically verified diseases or known genotypes. When comparing participants with a certain disease and healthy controls, information on which tests have been performed to verify the diagnosis should be given. The number of participants included is influenced by the design of the experiment and the requirements of the post-experiment statistical analysis. A prior power analysis will help to determine whether the number of participants included is sufficient to avoid Type I error. The health status of the participants should be reported; it is customary to do this in the form of inclusion and exclusion criteria. In the case of healthy volunteers, medication with potential influence on the pupillary responses should be excluded. Many drugs of different classes can affect the pupil, however, there are some general patterns. The most common offenders are drugs that interact with the sympathetic and parasympathetic innervations of the iris, either peripherally or centrally (Figure 4), and drugs that influence the level of arousal (63) due to the coupling between arousal and autonomic activity (64–66). Many drugs in overdose can induce non-specific effects, such as general CNS depression, leading to coma, or CNS over-excitation, leading to seizures. CNS depression is usually accompanied by miosis, and over-excitation by mydriasis. Table 2 gives an overview of selected topical and general medication potentially interfering with the pupillary responses. However, if patients are included, it may not be possible to exclude all these medication; in this case, all medication should be documented.

**Table 2:** Effect of drug treatment on the pupil.

**Drug**	**Mechanism**	**Pupil**
**TOPICAL**		
Pilocarpine	cholinergic	miosis^a^
Carbachol	cholinergic	miosis^a^
Aceclidine	cholinergic	miosis^a^
Atropine	anticholinergic	mydriasis^b^
Scopolamine	anticholinergic	mydriasis^b^
Tropicamide	anticholinergic	mydriasis
Phenylephrine	α_1_-adrenoceptor agonist	mydriasis
Methoxamine	α_1_-adrenoceptor agonist	mydriasis
Apraclonidine	α_1_-adrenoceptor agonist	mydriasis^c^
Dapiprazole	α_1_-adrenoceptor antagonist	miosis
Brimonidine	α_2_-adrenoceptor agonist	miosis^d^
Cocaine	noradrenaline uptake inhibitor	mydriasis
**SYSTEMIC**		
Antihistamines	H1 histamine receptor antagonists	miosis^e^
**ANTIHYPERTENSIVES**		
Prazosin	α_1_-adrenoceptor antagonist	miosis^f^
Clonidine	α_2_-adrenoceptor agonist	miosis^g^
**ANTIARRYTHMICS**		
Disopyramide	anticholinergic	mydriasis
**DRUGS FOR PARKINSON'S DISEASE**		
Anticholinergics	blockade of muscarinic receptors	mydriasis^h^
Dopaminergics	stimulation of D2 dopamine receptors	mydriasis^i^
**ANTIDEPRESSANTS**		
Tricyclic	mainly noradrenaline uptake blockade	mydriasis^j^
Reboxetine	noradrenaline uptake blockade	mydriasis
Venlafaxine	noradrenaline/serotonin uptake blockade	mydriasis
SSRIs	serotonin uptake blockade	no effect^k^
**ANTIPSYCHOTICS**		
Phenothiazines	α_1_-adrenoceptor antagonist, sedation	miosis^l^
Haloperidol	α_1_-adrenoceptor antagonist	miosis
**SEDATIVES**		
Benzodiazepines	GABA receptor agonist → sedation	no effect^m^
**PSYCHOSTIMULANTS**		
Amphetamine	noradrenaline releaser	mydriasis
Modafinil	dopamine uptake blocker	mydriasis^n^
**ANALGESICS**		
Opiates	stimulation of inhibitory μ receptors	miosis^o^
**ANTIEMETICS**		
Scopolamine	anticholinergic	mydriasis
**ANTI-INCONTINENCE DRUGS**		
	anticholinergic	mydriasis^p^

a *glaucoma treatment*.

b *myopia treatment*.

c *in Horner's syndrome (supersensitive α_1_-adrenoceptors)*.

d *drug reduces noradrenaline release (glaucoma treatment)*.

e *first generation antihistamines (e.g., diphenhydramine, cyclizine) penetrate into the brain where they block H1 histamine receptors, leading to sedation*.

f *drug blocks α_1_-adrenoceptors in vascular smooth muscle*.

g *drug stimulates inhibitory α_2_-adrenoceptors on central noradrenergic neurones, leading to sedation and sympatholysis*.

h *include orphenadrine, procyclidine, trihexyphenidyl*.

i *D2 dopamine receptor agonists (e.g., pramipexole) stimulate inhibitory D2 receptors on wake-promoting central dopaminergic neurones, leading to sedation. This is expected to cause miosis, however, paradoxically, pramipexole causes mydriasis [see (65)]*.

j *Tricyclic antidepressants block the uptake of noradrenaline, potentiating noradrenergic neurotransmission, and this would lead to mydriasis. However, they have some other effects: blockade of muscarinic cholinoceptors would lead to mydriasis and sedation, and blockade of α_1_ adrenoceptors would cause miosis. The overall effect reflects the balance between these actions: mydriasis due to noradrenaline uptake blockade and cholinoceptor blockade is counteracted by miosis due to α_1_-adrenoceptor blockade and sedation. This explains the variable effects of tricylic antidepresssants on the pupil: imipramine and desipramine dilate it, while amitriptyline has little effect on it*.

k *Selective serotonin reuptake inhibitors (SSRIs) block serotonin receptors in a complex network of serotonergic neurones associated with different excitatory/inhibitory receptors. The overall effect is little or no change in pupil diameter*.

l *These drugs (e.g., chlorpromazine, trifluoperazine) also have anticholinergic effects that would lead to mydriasis. However, α_1_-adrenoceptor blockade and sedation predominate, leading to miosis*.

m *Paradoxically, although the benzodiazepine diazepam is highly sedative, it has no effect on pupil diameter [see (66)]*.

n *Modafinil blocks dopamine uptake at exciatatory synapses on central noradrenergic neurones: this leads to increase in arousal and sympathetic activity*.

o *Stimulation of inhibitory μ receptors on central noradrenergic neurones leads to sedation and sympatholysis*.

p *These drugs (oxybutynin, festerodine) inhibit voiding of the urinary bladder by blocking cholinoceptors in the detrusor muscle*.

#### Adaptation State of the Retina

We recommend to report the background and room illuminance (Lux) during the measurements and, if applicable, the pre-dark or pre–light adaptation times to room illumination (light-adapted vs. dark-adapted vs. mesopic condition vs. no adaptation). With regard to dark adaptation times, Wang et al. showed a significantly increased transient and sustained contraction amplitude of the pupil light response during dark adaptation and consequently suggested a period of 20 min of dark adaptation for consistent pupil responses (67).

The first pupillary response in a series may be excluded from analysis, e.g., due to a larger response owing to the pre-stimulus state of relative dark adaptation. This has to be applied in a consistent way for all recordings and has to be reported in the methods.

#### Stimulus Characteristics

Particular importance is ascribed to the stimulus characteristics themselves to make an experiment transparent and comparable to others. These include the method of stimulus presentation (full-field stimulation vs. local stimulation) and, in the case of a local stimulation, the exact stimulus size.

Furthermore, information regarding the stimulus intensity, duration, inter-stimulus time and wavelength (color) should be reported. These parameters determine whether rhodopsin-driven rod responses, opsin-driven cone responses, or melanopsin-driven ipRGC responses are primarily elicited.

#### Baseline Diameter

For a reliable interpretation of the data and to facilitate replication of findings, the absolute pupil baseline diameter before the stimulation should be reported. This metric varies widely across participants with a characteristic decreasing pupil size with age (2) and hints for smaller pupil sizes in specific retinal diseases, e.g., in CNGA3-linked Achromatopsia (35). Further analyses should usually be based on relative values as pupillary responses are dependent on the initial baseline diameter, which should be obtained during a sufficiently long recording period to ensure a steady and reliable estimate. Such normalizations limit the effect of fluctuations in diameter and control for individual differences in pupil diameter, including senile miosis. To normalize pupillary responses, the absolute pupil diameter at any given time is converted to a relative pupil constriction amplitude in percent from baseline, e.g., by the following formula:

relative pupil constriction amplitude at time *x* = [(baseline pupil diameter – absolute pupil diameter at time *x)*/baseline pupil diameter] × 100.

For specific research questions, particularly in psychological experiments with additional behavioral or performance context and pharmacological studies, it might also be reasonable to evaluate the actual change in diameter; we discuss this issue in the specific chapters.

## II. Part: Specific Standards for Pupillography

Beside the above mentioned general standards that we strongly encourage researchers to consider in a publication, specific standards for the different research areas and applications of pupillography are important. In the following, the proposed specific standards and suggested investigation strategies regarding suitable stimulus characteristics as well as appropriate response analysis parameters are presented.

## 1. The Afferent Pupillary Pathway Authors: Carina Kelbsch, Andrew J. Zele, Beatrix Feigl And Helmut Wilhelm

**Figure 1:**
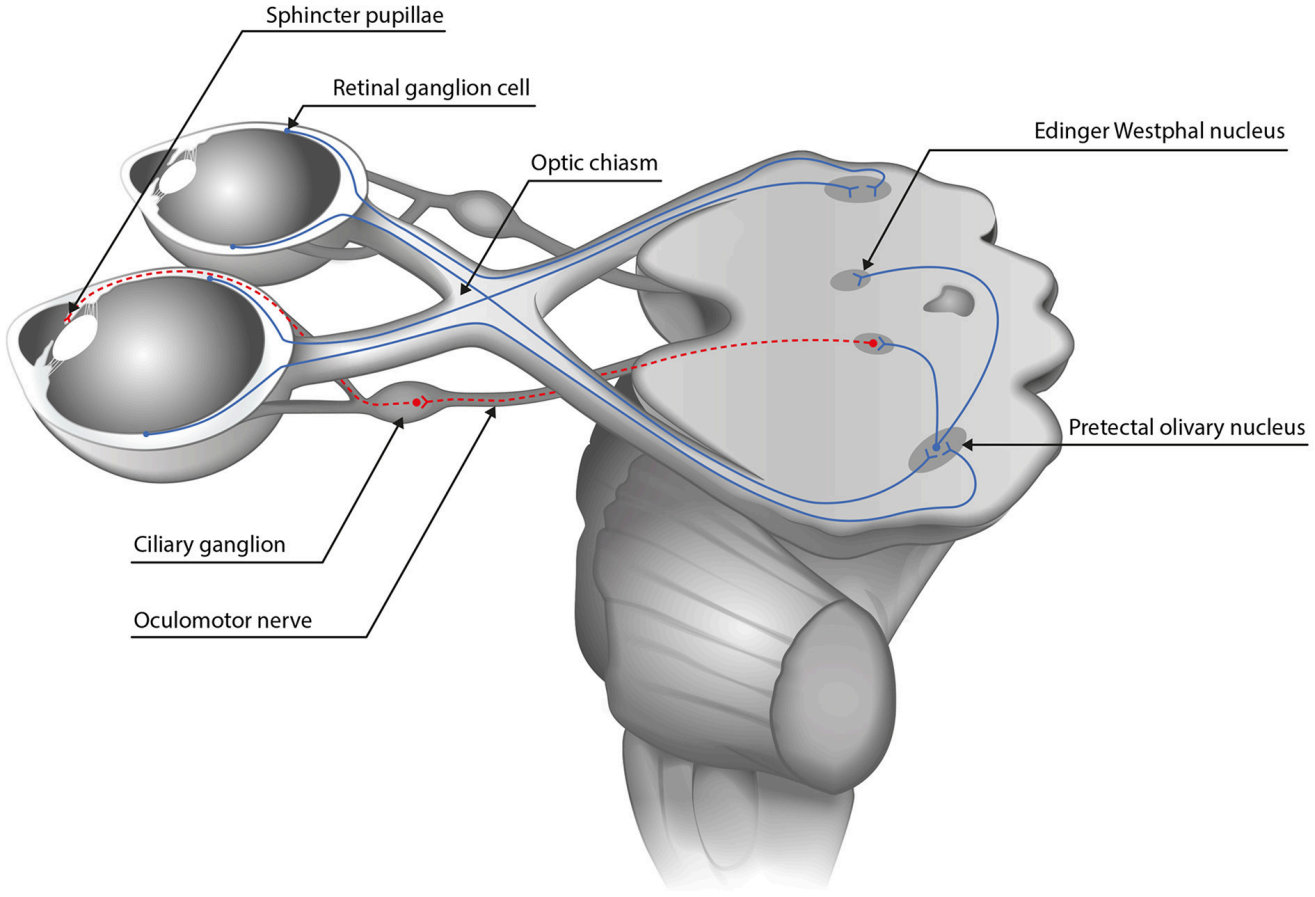
The pupillary pathway. The afferent pupillary pathway comprises the retinal photoreceptors, the bipolar cells and the retinal ganglion cells whose axons form the optic nerve. Temporal fibers run ipsilaterally while the nasal fibers cross to the contralateral side in the optic chiasm. Afterwards, they form the optic tract and synapse at the olivary pretectal nucleus therefrom connecting to both Edinger Westphal nuclei (blue continuous line). The efferent pathway from the Edinger Westphal nucleus to the pupillary sphincter via the ciliary ganglion is depicted in dashed lines.

The afferent pupillary pathway consists of the retinal photoreceptors, the bipolar cells, the retinal ganglion cells, the optic nerve and optic tract, ends at the olivary pretectal nuclei which connect to the Edinger Westphal nuclei where the efferent pathway begins (see Figure 1).

The following variables influence the pupillary light response and should therefore be specified:

- Stimulus wavelength (nm; peak and bandwidth at half maximum)
- Stimulus irradiance (log photon.cm^−2^.s^−1^, W.m^−2^), stimulus luminance (cd.m^−2^) and/or stimulus illumination (Lux)
- Stimulus size (degrees visual angle) and shape (if not circular; e.g., quadrant)
- Stimulus localization/ fixation eccentricity (if not full-field)
- Stimulus duration (s) and frequency (Hz; for periodic temporal modulation)
- Background wavelength and irradiance (log photon.cm^−2^.s^−1^) or luminance (if not dark)
- Dark and light adaptation times (min)
- Inter-stimulus interval (s)
- Number of repetitions

To assess retinal function, specifically designed stimulation paradigms are required to stimulate the extrinsic pathway via rods and/or cones or the intrinsic pathway of melanopsin-expressing intrinsically photosensitive retinal ganglion cells (ipRGCs). In the following, we first provide recommendations for test stimulation protocols and analyses for objectively quantifying rod and cone photoreceptor inputs to the afferent pupillary pathway. These are based on an evaluation of modern approaches that we anticipate can provide a platform to facilitate the development of new protocols (Chapter 1.1). Then, we introduce a series of recommendations for the assessment of melanopsin inputs to the pupillary pathway (Chapter 1.2).

### 1.1 Rod and Cone Photoreceptor Contribution to the Pupil Light Reflex

#### Introduction

Human vision spans more than ~10 log of units of retinal illumination through the combined activity of rod and cone photoreceptors. In bright, photopic illumination, vision is initiated by the output of three different cone classes with overlapping absorption spectra and peak sensitivities at short wavelengths [S-cones: ~445 nm (corneal, 10° standard observer); ~420–430 nm (retinal)], medium wavelengths [M-cones: ~541 nm (corneal, 10° standard observer); ~530–534 nm (retinal)] and long wavelengths [L-cones: ~567 nm (corneal, 10° standard observer); ~561–563 nm (retinal)] (68–71). Rod photoreceptors [peak ~507 nm (corneal); ~491–498 nm (retinal)] initiate vision under dim, scotopic illumination and both the rods and cones are operational at intermediate, mesopic illuminations. In addition to their different spectral sensitivities, the rod and cone systems show different temporal, spatial and adaptation responses, and topographical retinal distributions. Taken together, their unique and combined contributions to vision (and the pupil light reflex) will vary with the spectral, temporal, spatial and adaptation characteristics of the stimulus conditions (72) and so differences in the stimulus conditions will be reflected in changes in the relative sensitivity of the two systems and their contributions to the pupil light reflex. Ultimately, spectral sensitivity measurements will be necessary to quantify the relative rod and cone contribution to the pupil light reflex for a particular set of stimulus conditions and analysis metrics as has been demonstrated for the pupil constriction during light stimulation (73) and the post-illumination pupil response, or PIPR (8, 74, 75).

#### Stimulus Characteristics

The separation and measurement of rod and/or cone contributions to the pupil has been assessed using techniques pioneered in visual psychophysics. A primary approach uses selective chromatic adaptation (76) with monochromatic test lights presented against monochromatic adapting background lights of different irradiances. The idea is that a background wavelength and irradiance can be chosen to desensitize (adapt) one or more photoreceptor classes, with the test wavelength chosen to bias the response to another photoreceptor class. The rod system has a higher luminous efficiency (V'λ) at shorter wavelengths than the cone pathway (Vλ), with this difference approaching zero at longer wavelengths (>650 nm) (77). Below cone threshold (~1 Troland), all stimulus wavelengths are mediated via rods (note that melanopsin contributions to vision and the pupil are still to be defined under scotopic illumination, but are believed to be negligible). In the mesopic and moderate photopic range (below rod saturation), no monochromatic light will isolate rods or cones, with the relative degree of separation dependent on the sensitivity of the two systems to the stimulus conditions. When possible, this should be estimated.

To favor detection to the rod system, Aguilar and Stiles (78) determined that a blue-green test stimulus (<490 nm) will provide a high ratio of rod to cone sensitivity; a red adapting background light (>610 nm) stimulates the cone system more than rods (a low ratio of rod to cone sensitivity) and reduces cone sensitivity. The stimulus light also entered the eye at the edge of the pupil to take advantage of the Stiles-Crawford (79) effect. To bias detection to the cone system, high irradiance adapting fields are required to saturate the rods, with the monochromatic test and field wavelengths reversed. When assessing the cone system, the stimulus properties, particularly the irradiance, size, duration and retinal eccentricity will influence the responsivity of the three primary post-receptoral pathways (80, 81). The success of selective chromatic adaptation is also limited by the assumption that the rod and cone systems are independent (the duplicity theory of vision), and this is not the case due to the rod and cone signals sharing the same post-receptoral neural pathways (72). For cone mediated pupillary responses, a high irradiance adapting field becomes problematic, as it drives the pupil into a relatively miotic state, reducing its dynamic range of movement to superimposed pedestal light stimuli. Therefore, there is usually a compromise between the background adapting field intensity and the level of rod suppression when attempting to isolate cone mediated pupillary responses.

Given that age-related changes in the optical media attenuate the stimulus corneal irradiance to modify pupillary responses, lens density should be estimated and controlled for in the study design [e.g., LOCS III; (82)]. However, an increase in lens density may also be partially compensated for by photoreceptor adaptation. The absorption of the stimulus light at the test wavelengths can be quantified (83, 84). It's necessary to highlight that when pupillary responses are measured with rods and cones in different states of sensitivity, this influences comparisons about the degree of rod and cone photoreceptor dysfunction detected in patients. Moreover, retinal and/or optic nerve disease can lead to a remodeling of the neural pathways (85) and so the level of photoreceptor separation may be dissimilar within and between patients and healthy control participants. Inferences about the relative degree of rod and cone dysfunction in disease are presumably possible when the two systems are measured under similar viewing conditions. Current research addresses this issue by using multiple-primary colorimetric techniques with the method of silent substitution (86–90). With this approach, specific photoreceptor classes (e.g., rods, cones, melanopsin) can be directly modulated to study the afferent pupillary response; it is evident that the pupillary responses from different photoreceptor classes vary in amplitude and phase depending on the photoreceptor input combination and so in the future these findings will be important for developing new approaches to isolate and separate rod, cone and melanopsin contribution to the pupillary response.

There are examples of chromatic pupillometry methodologies that provide initial efforts to separate rod and cone function through the careful control of the wavelength, irradiance, size and duration of the test stimuli; the degree of separation of rod and cone (and melanopsin) function that these conditions provide is still to be determined. At light levels below cone threshold, short wavelength lights are presented in the dark to bias the response to rods, with the PIPR amplitudes minimized under such conditions (9, 11, 35). To ensure maximal rod sensitivity, the pre-stimulus dark adaptation time should be at least 30 min (91); although this is not practical for all clinical protocols, shorter periods will influence the relative rod and cone sensitivity to the test stimuli. When using selective chromatic adaptation to bias pupil responses to the cone system, a red test stimulus (>610 nm) is presented against a blue background (< 490 nm) to suppress rod function (11); a 467 nm, 0.78 log cd.m^−2^ background has a similar scotopic luminance to a 30 cd.m^−2^ white background as used in the standard ISCEV protocol (11, 92). Quantal matched long (and short) wavelength test stimuli can be included in each condition as a control. Because the pupil diameter returns to the dark-adapted baseline faster than after photopic test stimuli, the inter-stimulus interval is shorter for scotopic test conditions. As mentioned previously, a bright adapting light can present its own problem with reducing the dynamic range of pupil movement due to the relatively miotic state induced by a bright adapting background. Furthermore, in light-adapted condition, pupillary measurements become noisier as light-induced oscillations may occur during the exposure to the background light (2).

#### Analysis

**Figure 2:**
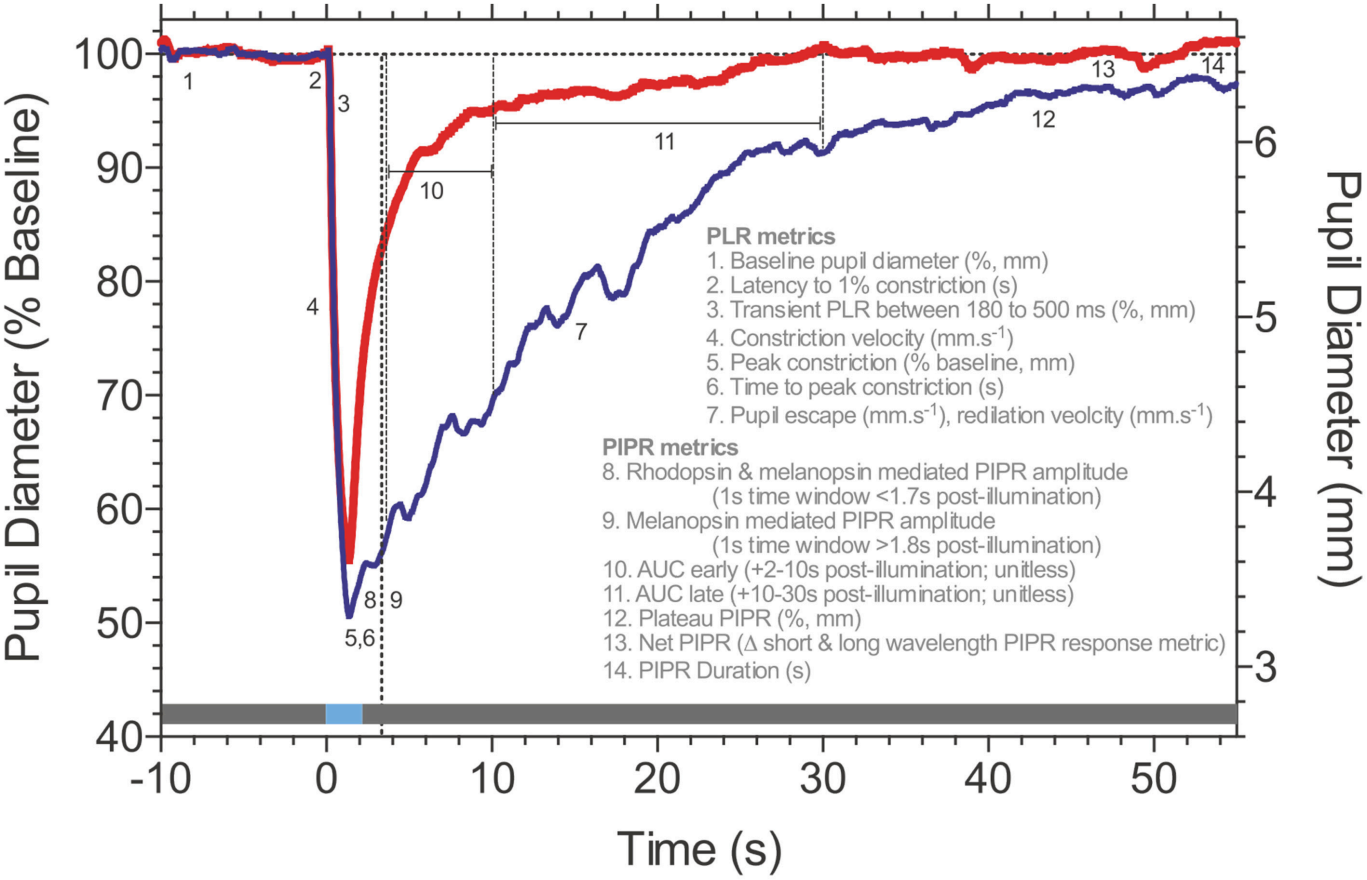
Post-Illumination Pupil Response (PIPR) metrics. Consensual pupillary response to 1 s pulses (horizontal blue line at time 0; 465 nm blue, 637 nm red-the gray line represents the pre- and post-stimulus periods in the dark) measured in Maxwellian view (35.6° diameter stimulus; 15.1 log quanta.cm^−2^.s^−1^). Details of the pupil light response (PLR) and Post-Illumination Pupil Response (PIPR) metrics are described in the figure. Data are for a representative healthy observer (traces are the average of 3 repeats). Traces courtesy of Prakash Adhikari, Beatrix Feigl and Andrew J. Zele.

During presentation of a light stimulus with low melanopsin excitation, the pupil light reflex is mainly driven by extrinsic cone and rod inputs to ipRGCs. For these conditions, analysis metrics include the transient response, latency to constriction and maximum pupil constriction amplitude. Both, absolute (in mm) or relative amplitudes (in %) relative to the baseline pupil diameter can be used, but the relative pupil constriction amplitude should always be provided (see Part I, general standards). Another parameter is the maximal constriction velocity which is proportional to the amplitude. Additional information may be gained by measuring the latency to constriction, inversely correlated to stimulus brightness and size and the time to maximal constriction. For an overview of typical pupillographic analysis metrics as well as more detailed information regarding stimulation characteristics, please refer to the following chapter (Figure 2; 1.2 Melanopsin-The Post-Illumination Pupil Response, PIPR).

#### Application

Chromatic pupillometry has been applied in various forms in clinical studies, including those with patients with rod and cone dystrophies such as Retinitis Pigmentosa (9, 24–27, 93, 94) and achromatopsia (35, 95), as well as in animals, including canine (96, 97) and mouse (98). These paradigms quantify the PLR metrics (see also Figure 2; 1.2 Melanopsin-The Post-Illumination Pupil Response, PIPR) after exposure to test stimuli specified according to their:

- stimulus wavelength (e.g., narrow band chromatic lights, broadband white lights), duration, area, fixation eccentricity and the dark- and light-adaptation levels (and pre-adaptation durations) that are optimized to stratify the rod-cone cut-offs under conditions of dark and light adaptation (11, 35, 99–103) that bias pupil responses to either rods or cones based on their characteristic spatial and temporal summation (104). A rod-favoring-condition may include a dim, short wavelength stimulus (e.g., 4 ms; 0.01 Lux corneal illumination) after prolonged dark adaptation and cone-favoring-condition with a brighter long wavelength stimulus (e.g., 1000 ms; 28 Lux) after a 10 min period of light adaptation (35);
- increment pulses increasing in a step-wise pattern from high mesopic to low photopic luminances (1, 10, 100 cd.m^−2^; 45° diameter stimuli), which may be followed by a 30 s dark-period for additionally recording the Post-Illumination Pupil Response (95);
- a logarithmic increase in stimulus irradiance from 8.5 to ~14.5 log quanta (scotopic to photopic; full-field Ganzfeld stimuli) over a 2 min period, with 1 min pre- and post-stimulus periods of darkness (23);
- measurement of the peak-to-trough amplitude of the flicker (0.5 Hz) pupil response to blue test stimuli (with high melanopsin excitation) and red test stimuli (with low melanopsin excitation) (105), with the amplitude indicative of the level of interaction between the outer retina photoreceptors and inner retinal melanopsin, as calculated using the phase amplitude percentage (PAP) metric (28) that has application in disease detection (29, 44).

### 1.2 Melanopsin-The Post-illumination Pupil Response (PIPR) Authors: Andrew J. Zele, Beatrix Feigl, Yanjun Chen, Paul D. Gamlin and Randy Kardon

#### Introduction

In human and non-human primate retina, melanopsin-expressing intrinsically photosensitive Retinal Ganglion Cells (ipRGCs) stratify the inner and outer regions of the inner plexiform layer, encircle the foveal pit, and increase in dendritic field diameter with increasing eccentricity, independent of their soma size (7, 16, 106). Signals originating in outer retinal rod and cone photoreceptors are transmitted extrinsically to ipRGCs via synaptic connections with DB6 diffuse bipolar cells and dopaminergic amacrine cells (16, 107–109). With a morphology and functionality distinct from conventional retinal ganglion cells (7), ipRGCs project 1) via the retinohypothalamic tract to multiple brain regions (110) for non-image forming functions including to the suprachiasmatic nucleus, the endogenous biological clock, to synchronize biological and physiological processes to the 24-hour light-dark cycle (5, 6, 111–115), 2) the pretectal olivary nucleus in the midbrain to regulate pupil diameter (8, 110), and 3) the lateral geniculate nucleus of the thalamus (7, 16, 110) for image forming visual functions (90, 116–118).

A signature biomarker of human melanopsin function is the Post-Illumination Pupil Response (PIPR), the sustained pupil constriction after light offset (Figure 2). This PIPR follows a characteristic irradiance-response relationship (8, 11, 74) with a half-maximal constriction for a retinal irradiance of ~13.5 log photons.cm^−2^.s^−1^ (8); the largest sustained pupil constriction occurs at ~482 nm, the peak sensitivity of the melanopsin photopigment, as evidenced directly from spectral sensitivity measurements of the PIPR in humans (8, 28, 74) and non-human primates (8). Between light offset and ~1.7 s post-illumination, the peak sensitivity of the PIPR shifts to longer wavelengths to reflect major inputs from rhodopsin and melanopsin, with minor cone contributions (75).

#### Stimulus Characteristics

The following stimulus optimizations pertain to the measurement of the melanopsin-mediated PIPR measured in a darkened environment without immediate pre- or post-stimulus light adaptation. The optimal stimulus wavelength is nearer the melanopsin peak sensitivity (~482 nm), but any wavelength can produce PIPR amplitudes similar to the optimal wavelength by suitably scaling the irradiance according to the principle of univariance (119). Such alternate wavelength selections can be advantageous e.g., for limiting confounds from age-related lens attenuation. A long wavelength light (e.g., >635 nm) is typically included as a control to quantify non-specific autonomic factors, to measure extrinsic photoreceptor inputs to the pupil under conditions to which melanopsin has low sensitivity and to rule out the effect of lens attenuation.

Light output is ideally specified as the corneal or retinal irradiance using radiometric units (e.g., photon flux [log photons.cm^−2^.s^−1^] or irradiance [W.m^−2^]). Stimuli of different wavelengths need to apply the same irradiance for comparability. Radiometric units are preferred because the photopic relative luminous efficiency (V) is defined exclusively in terms of additive L+M cone function (120). The quantification of the light in terms of its melanopsin excitation, that is the alpha-opic lux (121) or relative cone Trolands (122) will facilitate comparison between radiometric and photometric units.

Luminance (cd.m^−2^) or corneal illumination (Lux), which are widely used in clinical applications, need to be combined with the stimulus wavelength.

Stimulus areas can be custom-selected to be full-field (e.g., Ganzfeld) or smaller focal-fields that localize responses to select visual field regions. The pupil pathway is presumed to integrate over larger retinal areas than image-forming vision (102, 123–126) and pupil measurements indicate that the PIPR follows a hill-of-vision with larger amplitudes in central than peripheral retina (127) which could be attributed to eccentricity related changes in ipRGC dendritic field density (7, 16, 106). Therefore, the select spatial stimulation of the PIPR will have advantages in the detection of early retinal dysfunction (21) due to the reduced number of ipRGCs [~3,000; (7)] compared to conventional ganglion cells [~1.5 million; (128)]. The PIPR has been assessed using stimulus durations ranging from 4 ms to 30 s (11, 74, 102), with 1 s test pulses (Figure 2) showing wide applicability due to their large, robust and repeatable PIPR amplitudes that are sustained for about 80 s with stimulus irradiances above 14 log photons.cm^−2^.s^−1^ (74). The PIPR duration should be considered when determining the inter-stimulus interval, as well as the recovery from after-images of the stimulus light. The PIPR is typically measured using increment pulses. The flicker pupillary response to sinusoidal test stimuli has a low pass characteristic with a peak amplitude at ~0.5 Hz and high frequency cut-off near approaching 9 Hz (86, 105, 129, 130). With such sinusoidal temporal modulations the PIPR amplitude is presumed to be dependent on stimulus irradiance, and independent of temporal frequency in the range of 0.2–4 Hz (105).

A natural pupil is subject to fluctuations in diameter due to variation in autonomic nervous system tone, accommodation and vergence eye movements, environmental factors (ambient light, sounds, etc.) and interval (attention and alertness that reflects central nervous system adrenergic outputs) (131). A problem in closed-loop pupillographic paradigms is that the pupil of the test eye changes size during light presentation and subsequently alter the retinal irradiance (11, 132, 133). This could be overcome by mydriasis (in consensual pupil recordings) or using an open-loop Maxwellian view pupillometry system (with or without mydriasis) that focuses the stimulus image within the plane of the pupil (134). However, in clinical trials both methods may rarely be practicable because mydriasis interferes with other essential ophthalmological tests and not all laboratories have access to Maxwellian view systems.

#### Analysis

Post-illumination pupil response metrics quantified from 1.8 s post-stimulus onwards in time will provide a direct measure of human melanopsin function (Figure 2), with variability being the key determinant for the particular choice of metric (74); analyses are conducted with reference to the pre-stimulus baseline pupil diameter recorded prior to stimulus onset to achieve a stable and robust estimate in millimeters (mm, absolute pupil diameter) or percentage (%, relative pupil constriction amplitude). Under light adapted conditions, the pupil receives significant melanopsin input (88). Commonly implemented PIPR metrics include the plateau PIPR (8, 9); the PIPR amplitude [e.g., a 1s window at a pre-set time, such as 6 s post-illumination; (11, 74)] that is set for a participant cohort and specific stimulus conditions by determining the largest difference between the long wavelength (control) and short wavelength (test) PIPR amplitudes for the control group during a moving 1 s window (44); the PIPR average during pre-specified time epochs including the early and late Area Under Curve (AUC) [e.g., 2–10 s or 10–30 s post-illumination; (26, 135, 136)]; the net PIPR is the difference between the long wavelength (control) and short wavelength (test) PIPR amplitudes (19, 137); the redilation velocity (20) and the PIPR duration (74). Of these metrics, the PIPR amplitude and plateau PIPR show the lowest coefficient of variation which indicates these two metrics are the most reliable from one test to another in the same participant (78). During presentation of a light stimulus with low melanopsin excitation, the pupil light reflex is mainly driven by extrinsic cone and rod inputs to ipRGCs and metrics such as the transient response, latency to constriction and maximum pupil constriction amplitude are considered for analysis of outer retinal function (95). IpRGCs also act to keep the pupil constricted during light stimulation (73, 138, 139). The Phase Amplitude Percentage (PAP) can be used to study the interaction between inner and outer retinal inputs to the phasic pupil response during sinusoidal light stimulation (28).

#### Application

IpRGCs are presumed to be relatively robust to aging, with functional studies showing stable PIPR responses into the seventh decade (137, 140), and histological studies of human retina showing ipRGCs density is stable until this age, with a loss in density and dendritic arborization of human ipRGCs after the age 70 (141). The stability of PIPR amplitude across much of the lifespan (after controlling for age-related lens attenuation) makes it an objective reference marker of ophthalmic function for applications in clinical aging studies. No effect of refractive errors ranging between +3.00 and −9.25 D on the melanopsin mediated PIPR amplitude could be shown (140).

The melanopsin mediated PIPR can be applied in clinical cohorts to detect and monitor the progression of ipRGC dysfunction in disease. IpRGC dysfunction has been observed in a variety of retinal and optic nerve diseases, in particular at early stages, including in glaucoma (19–23), diabetic retinopathy (31, 32, 142), age-related macular degeneration (29, 30), and ischemic optic neuropathy (143). IpRGC function is largely preserved in mitochondrial optic neuropathy (135, 144) and in Retinitis Pigmentosa (9, 24–27). Altered melanopsin-dependent pupillary responses are also evident in neurologic and psychiatric conditions including seasonal affective disorder (41, 45), multiple sclerosis (145) and Parkinson's disease (44). As a direct measure of melanopsin function, the technique also has widespread application in the assessment of ipRGC function in chronobiology (37, 39).

### 1.3 Special Clinical Applications

#### Pupillographic Swinging Flashlight Test

Examination of the afferent pupillary pathway is a routine test in clinical and basic science investigations to determine the functionality of the retina and optic nerve signaling to the brain in the healthy eye or in specific disease. The comparison of the pupil light response between both eyes with the so-called swinging flashlight test is the standard for screening and diagnosing unilateral or asymmetric neuroretinal deficits by revealing a relative afferent pupillary defect (RAPD). The swinging flashlight test was first described by Levatin (146) and further developed by Thompson (147), and can be either assessed by an experienced clinician using a flashlight and neutral density filters, or by using pupillography that compares the pupil constriction amplitudes of both eyes quantitatively. With automated pupillography a stimulus response curve can quantify the difference between both eyes objectively (148) as opposed to the manual swinging flashlight test that is subjective and introduces examiner bias. The amplitude of the pupillary response is proportional to the logarithm of the intensity of the test light. Assessing the RAPD automatically might particularly help in precisely monitoring possible therapeutic effects in optic nerve diseases or serve as a screening method for defects of the afferent pathway. Pupillographic and swinging flashlight evaluation with neutral density filters of the RAPD have been performed e.g., in glaucoma (149–151) showing that the severity of RAPD correlates with the magnitude of field defects. However, diseases of the afferent visual system do not necessarily equally affect the results of perimetry or the RAPD (152).

The pupillographic swinging flashlight test should test the entire afferent pathway, and a white, full-field stimulus is recommended (151). The optimum stimulus brightness should constrict the pupil by approximately one third of its diameter. Stimulus length can be in accordance with the manual clinical swinging flashlight test that is usually between 1 and 3 s. The inter-stimulus interval should have at least the same length to allow the pupil's redilation, i.e., equal pupillary baseline conditions should be ensured for all stimuli. Fluctuations of pupil size or physiological anisocoria may influence the measurement. The best approach is therefore to measure both pupils simultaneously. Through this approach, the direct, bilateral pupil reactions to light, and the direct and consensual unilateral pupil reaction to light can be compared. At least four repeat measurements are recommended using pupillography as it is with the manual swinging flashlight test. This eliminates the problem of short term fluctuations of pupil size. Using a Maxwellian view condition (see also Chapter 1.2. Melanopsin) overcomes the fluctuating pupil size and hence irregular retinal irradiance. However, Maxwellian view is not always available in a clinical practice and does not eliminate fluctuations within the sympathetic system causing variations of the constriction speed and amplitude. It is therefore inevitable to repeat stimulus presentation several times to assess the afferent visual system. Adaptation before testing has to be considered: dark adaptation enhances the pupil light response while light adaptation will attenuate it. It is therefore mandatory to provide equal background illumination and equal stimulation length and brightness for both eyes but also an equal inter-stimulus interval.

#### Full-Field Pupillography, Pupil Perimetry/Campimetry

Pupillography is not only useful for the determination of an intact afferent limb of the pupillary pathway but can be performed to determine retinal functionality in certain retinal diseases. There are two strategies, either full-field pupillography to assess the entire neuroretinal function [e.g., in Retinitis pigmentosa: (24–26), or CNGA3-linked Achromatopsia: (35)], or pupil perimetry (stimulus presentation on a hemisphere) / campimetry (stimulus presentation on a flat monitor) to assess focal neuroretinal defects (94, 153–155).

When using focal stimuli, pupil visual field maps can be derived (156). Stimulus size may vary between large hemifields to small 1° stimuli to map visual fields objectively. Stimuli are classically presented one after another or using a multifocal pattern strategy (33, 157, 158). Ideally, a stimulus has to be large or bright enough to elicit a reliable pupil response but should avoid causing stray light. A challenge in pupil perimetry can be unstable fixation. Therefore, stimulus length is usually shorter (around 200 ms) in pupil perimetry/campimetry than the stimulus length used in pupillographic swinging flashlight testing (1–3 s) to avoid the patients wandering eyes during stimulus presentation. Nevertheless, fixation is essential in perimetric strategies for an accurate stimulus presentation on the retina, thus a gaze-controlled strategy (e.g., via eye-tracking) is recommended, as it allows for a retinotopic stimulation regardless of fixation problems (153). As pupil measurements are objective, pupil perimetry/campimetry also helps in distinguishing real visual field defects from functional visual field loss and malingering (153–155).

Each laboratory using pupil perimetry/campimetry needs to establish normative values or use a commercially available device with an existing database of normative (age corrected) values. This is not only important in a clinical setting but also for basic science evaluations of the pupil.

## 2. The efferent pupillary pathway Author: Helmut Wilhelm

### Introduction

The efferent pupillary pathways comprise the cholinergic pathway to the sphincter muscle and the adrenergic pathway to the dilator muscle of the iris. The cholinergic pathway begins in the dorsal region of the oculomotor nucleus complex (159). It runs with the third nerve through the cavernous sinus via the ciliary ganglion where the second order neurons, named short ciliary nerves, begin. Those reach the sphincter muscle through the subchoroidal space. Nerve fibers supplying the ciliary muscle underlying accommodation run together with the pupillomotor fibers. The sympathetic pathway begins in the hypothalamus, projects down the brainstem to the centrum ciliospinale at the level of Th1–Th3, follows the sympathetic chain to the superior cervical ganglion where the terminal neurons innervating the dilator muscle start (160). Their axons run without synapse through the ciliary ganglion and are called long ciliary nerves.

### Stimulus Characteristics and Analysis in Clinical Applications

Pupillographic examinations of the efferent pupillary system have mainly the purpose to detect pathologies like oculosympathetic paresis (Horner syndrome), oculomotor nerve palsy, and tonic pupil (damage of the ciliary ganglion). Those diagnoses are usually based on clinical observation and pharmacological testing (cocaine or apraclonidine in Horner syndrome (161–165) or dilute pilocarpine in tonic pupil (166). Pupillography is not necessary to establish a reliable diagnosis. However, it may be helpful to distinguish Horner syndrome from simple anisocoria or other causes of anisocoria in so far that it can help to decide whether pharmacological testing is necessary or not (167).

#### Horner-Syndrome

**Figure 3:**
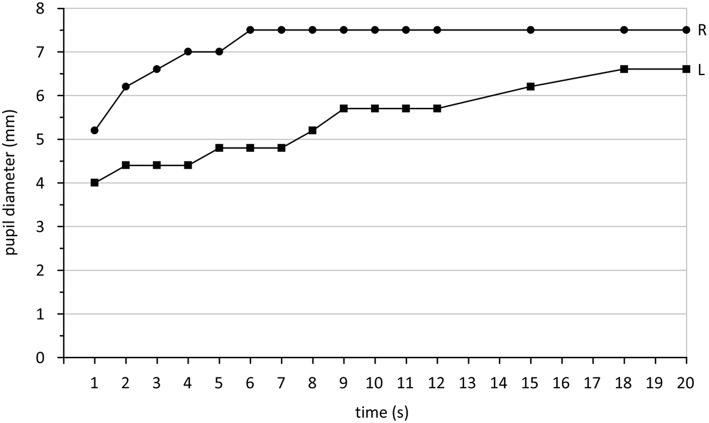
Pupil diameter (mm) measured in darkness after switching off a light stimulus over a time period of 20 s. The right eye (R) shows the typical quick redilation behavior of a healthy pupil while the left eye (L) reveals a dilation lag, typical for Horner syndrome. Data are taken from a patient with Horner syndrome in the left eye collected during standard care.

In Horner syndrome, pupil dilation is slowed down. Because this condition is, with very few exceptions, unilateral, comparison of the dilation behavior of both pupils is the best approach. Clinical studies establishing cut-off values are not available. A video study revealed the amount of anisocoria 4 s after switching off the light as the best parameter to diagnose Horner syndrome (168). It is recommended to use a bright stimulus to achieve a maximal possible pupillary constriction and then abruptly switch off the light and record the pupil behavior (169). A suitable parameter describing dilation is the -redilation time in comparison between both eyes (169). This is the time between maximal constriction and the time point when of the constriction amplitude has been lost by redilation. The constriction amplitude is defined as the difference between baseline and pupil size with maximal constriction. Another possibility is to measure constriction speed or post-illumination response as described in the chapter about intrinsic photosensitive ganglion cells (Chapter 1.2). Which approach would best distinguish Horner syndrome from physiologic anisocoria has not yet been studied. Using dilation lag based on the measurement of the redilation time, a sensitivity of 70% and a specificity of 95% for diagnosing Horner syndrome by pupillography is possible (169). Figure 3 shows the typical dilation behavior of a Horner pupil.

Independent from the parameters chosen, at least 3 tests per eye are necessary because pupil responses may vary and dilation lag might sometimes be detectable and sometimes not (170). Because a sympathetically denervated pupil dilates very slowly, it is recommended to extend the recording by at least 10 s or better 15 s after maximal constriction. Cocaine or apraclonidine testing (in children <1 year only cocaine) decides finally if Horner syndrome can be diagnosed.

#### Oculomotor Nerve Palsy and Neurological Emergency

In oculomotor nerve palsy, the accompanying outer eye muscle palsies determine the diagnosis. Oculomotor palsy limited to the pupil is extremely rare (171) and pupillography cannot contribute to the diagnosis. However, in the setting of raised intracranial pressure or uncal herniation or any other neurological emergency, pupillary light response is used to monitor the patients. There have been attempts to use pupillography instead of simple clinical observation to detect a light response (172). Indices based on pupillography have been used, but it is not yet clear if pupillography adds information additional to simple observation. By means of pupillography it may be easier to recognize a residual pupillary constriction in an emergency setting. By looking at the pupillogram it may be easier to decide whether a pupil has reacted to light or a random movement has been observed. The use of maximally bright light and at least 10 recordings per eye are recommended. Binocular recording has the advantage that afferent and efferent defects may be distinguished.

#### Tonic Pupil

The diagnosis of a tonic pupil is based on its clinical picture, reduced or absent response to light, preserved but slow near response and slow redilation. If the pupil is examined under magnification, small spontaneous segmental constrictions of the sphincter become visible (173). Also during near reaction, it may be observed that different parts of the sphincter react with different speed. The pupil is mostly not absolutely round but elliptically distorted. 0.1% pilocarpine constricts a tonic pupil and has less effect on a normal pupil.

It is possible to record both light and near response pupillographically (174). Constriction speed and amplitude are relevant parameters. By comparing to normal subjects, criteria for the diagnosis of a tonic pupil may be defined. This is especially helpful when diagnosing a bilateral condition (174). It can of course be used for precise measurement of pupillary diameter before and after pharmacological testing.

#### Diabetic Autonomic Neuropathy

Pupillary abnormalities in patients with diabetes have been found indicating sympathetic and parasympathetic dysfunction in comparison to healthy controls. While Dütsch et al. (175) could not reveal a difference between diabetes patients with and without cardiac autonomic neuropathies or peripheral neuropathies, Lerner et al. (176) found hints for reduced baseline pupil diameters and constriction amplitudes in patients with diabetes-related cardiac autonomic neuropathy compared to those without cardiac autonomic neuropathies. Consequently, when examining pupillary responses from a diabetes patient cohort, it is important to consider that they are not consequences of an underlying efferent neuropathy.

Although the diagnosis of efferent pupillary defects is a domain of clinical observation and pharmacological testing, pupillography might be a valuable supplement. It is important to be aware that any defects in the efferent pupillary pathway may change pupil movements and thus confound the interpretation of the pupil-based test in the assessment of the afferent pupillary pathway. For example, the pupil constriction in a pseudophakic eye may show a slower direct response (due to perturbation of the iris mechanics from the cataract surgery) thus leading to a misperception reduced pupillary response to light. Using consensual pupillary responses may provide a more precise measurement of the integrity of the afferent pathway if the efferent pupillary defect is a concern in the studied eye.

## 3. Pharmacology Author: Elemer Szabadi

### Introduction

**Figure 4:**
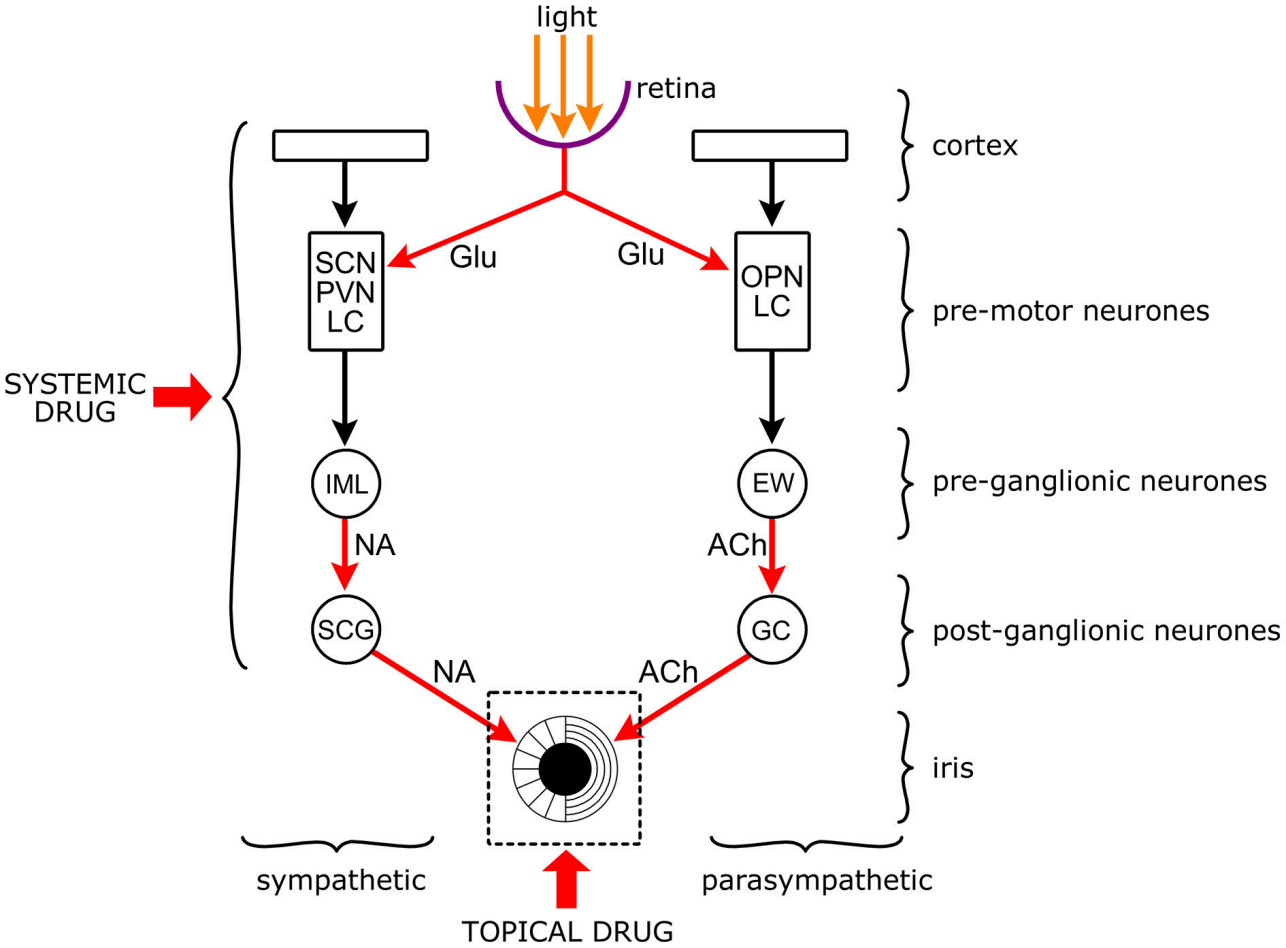
Targets of drugs in the neuronal network controlling the pupil. The pupil is an aperture in a diaphragm, the iris. The size of the pupil reflects the interaction between the circular sphincter muscle and the radial dilator muscle. The sphincter receives a parasympathetic and the dilator a sympathetic output. Both autonomic outputs consist of serially linked preganglionic and postganglionic neurones that are under the influence of premotor autonomic neurones. The premotor neurones channel the influence of other brain structures (e.g., cortex) and light to the preganglionic neurones. Premotor neurones: SCN: suprachiasmatic nucleus (hypothalamus); PVN: paraventricular nucleus (hypothalamus); LC: locus coeruleus (brainstem: pons); OPN: olivary pretectal nucleus (pretectum). Preganglionic neurones: IML: intermediate lateral column (spinal cord); EWN: Edinger-Westphal nucleus (brainstem: midbrain). Postganglionic neurones: SCG: superior cervical ganglion; GC: ciliary ganglion. Arrows are neuronal connections, red arrows are excitatory connections with identified neurotransmitters (Glu, glutamate; NA, noradrenaline; Ach, acetylcholine). Drugs can be applied topically to the surface of the cornea to affect the iris and the noradrenergic and cholinergic neuro-effector junctions, or systemically when they can affect any part of the central neuronal network. It should be noted that topically applied drugs may get into the systemic circulation leading to systemic effects, and systemically applied drugs may also affect the iris directly.

The anatomical and physiological features of the pupil make it eminently suitable for pharmacological studies. Its size (measured as diameter, or occasionally as area) is determined by the balance between two opposing smooth muscles in the iris that receive opposing sympathetic and parasympathetic innervations (Figure 4).

The two serially connected pre- and postganglionic neurons are under the influence of a network of premotor autonomic neurons in the brainstem and diencephalon which channel all physiological and psychological stimuli, including the effect of light, to the pupil. Changes in pupil diameter brought about by these stimuli, including drugs modulating them, are directly available to visual and instrumental inspection, recording, measurement and quantitative analysis. Furthermore, the iris is accessible to topically applied drugs creating, together with the concomitant recording of pupillary changes, a unique *in situ/in vivo* pharmacological test system.

Not surprisingly, pharmacological studies of the pupil are abundant, both in humans and non-human animal species. The use of drugs can help in unraveling the central neuronal network controlling the pupil, and can also provide valuable information about the drugs themselves by establishing their effects in a well-defined physiological/pharmacological system. Reports on the effect of drugs on the pupil require documentation of parameters of light stimulation and method of recording, like in any other field of pupillography, together with information on the pharmacological aspects of the study (characteristics of the participants and drug(s) used, design, measurement of drug effects, data analysis). It is important that all methodological detail is provided not only to help the reader to evaluate the study but also to help further investigators to replicate the study.

In this chapter, we propose some guidelines that should be adhered to when publishing the effects of drugs on the pupil. It is hoped that adherence to these guidelines would help the reader to better evaluate the study and facilitate replication. These guidelines relate to the study of human participants. However, many of them are also applicable to the study of non-human subjects.

### Specification and Stimulus Characteristics

#### Participants

If the study involves topical drug application, in addition to general information as number, age and sex, the color of the iris should be specified since pigment in the iris binds the applied drug leading to a reduction in the response (177).

#### Drugs

##### Topical application

A major issue in case of topical application is bioavailability of the drug that is largely determined by penetration through the cornea (178). Drugs can be applied to the surface of the eye in different forms (179). For pharmacological studies, drugs are used in aqueous or oily solutions. The formulation of the drug should be specified: it should be made clear whether the drug is used as a base or a salt. The vehicle should be specified: penetration through the cornea is usually better from oily solutions (180). Although the possibility of applying drugs to the surface of the eye as a continuous superfusion has been explored (181), the “blob” application in the form of eye drops has remained the common form. A calibrated micropipette should be used to apply a standard volume of solution (e.g., 10 μl) into the conjunctival sac. The molar concentration of the drug should be specified, together with the pH of the solution. It should be made clear whether any “penetration enhancer” [e.g., a local anesthetic; see (182)] has been used. Although topical application assumes that the effect of the drug is restricted to the eye to which the drug was applied, occasionally systemic effects can occur, affecting the fellow eye, and /or other parts of the body (183).

##### Systemic application

Drugs are usually administered orally, however, occasionally parenteral administration (e.g., infusion) is used (184). The formulation (base vs. salt) should be specified. Dosage per single oral dose, or concentration in infusion fluid and rate of infusion, should be specified. In single dose experiments pharmacokinetic evidence is needed to make sure that measurements coincide with the peak blood concentration of the drug.

#### Design

The design can vary according to the questions to be answered. It should aim at eliminating bias and contamination by procedural factors (e.g., practice effects). Therefore, it is common practice to use a double-blind design, and to allocate participants to sessions and treatments according to a balanced cross-over design. The index treatment should be compared with positive (i.e., a known treatment with the expected effect) and negative (placebo) **controls**. In the case of topical application, the fellow eye should receive treatment with artificial tear (i.e., placebo). However, if the measurements are taken in light, the response to the topical drug cannot be taken as the size of the drug-induced anisocoria, due to the operation of a consensual interaction between the pupils (185). Therefore, measurements should either be taken in darkness, or the response should be measured from the pre-treatment baseline in the index eye.

Apart from using positive and negative control treatments, it is also necessary to include a number of **collateral measurements** with expected effects in the relevant area. For example, if the potential sedative effect of a drug on the pupil is studied, non-pupillary effects of sedation can be included in the design [e.g., battery of visual analog scales, critical flicker fusion frequency; see (66)], or when potential sympatholytic or sympathomimetic effects on the pupil are investigated, non-pupillary sympathetic effects can be incorporated [e.g., changes in blood pressure and heart rate: see (186)]. The collateral evidence is important in corroborating the genuineness of the pupillary findings.

### Recording of Pupillary Effects of Drugs

#### Recording in Darkness

Infrared technology allows recording pupil diameter changes in darkness. Although some limited information may be obtained by studying the effects of drugs on **resting pupil diameter** in the dark, more comprehensive information can be gained by investigating their effects on light-evoked pupillary function (see below). Spontaneous **pupillary fluctuations** in the dark are recorded using the Pupillographic Sleepiness Test (PST). The PST and the specific standards for its use are discussed later in this paper (see Chapter 5). This test is amenable for the detection of the sedative and alerting effects of drugs, and its two quantitative indices (Pupillary Unrest Index and total power of fluctuations) correlate well with non-pupillary measures of the level of arousal (66).

#### Recording in Presence of Light Stimulation

For pharmacological studies both static (resting pupil diameter) and dynamic (pupillary reflexes) pupillometry can be used. The methodological requirements for light stimulation are the same as for other pupillographic investigations and are described in detail in the general standards section.

For pharmacological studies, it is desirable to study the effects of drugs on **resting pupil diameter** at a number of luminance levels, for several reasons. Firstly, in this way we obtain a much larger data set that would yield greater statistical power. Secondly, light can set the baseline at different levels that in turn would be reflected in the size of the responses, a lower baseline favoring dilator responses and a higher baseline constrictor responses (187). It should be noted, however, that apart from its mechanistic effect of setting the baseline, light also has a more specific effect in the case of sympathetic drugs, potentiating sympatholytic and antagonizing sympathomimetic effects (188).

The pupillary light reflex is evoked by a brief light pulse and the darkness reflex by sudden withdrawal of illumination. For pharmacological studies, the **light reflex response** is divided into two parts, latency and amplitude reflecting parasympathetic activation, and recovery time sympathetic activation (189). The parameters of the **darkness reflex response** (initial velocity, amplitude) are indices of sympathetic activation (66). For the light reflex response, it is recommended to use a range of stimulus intensities: this would enable the construction of light intensity/amplitude, light intensity/latency and light intensity/75% recovery time curves. The large dataset obtained in this way yields enhanced statistical robustness.

### Analysis

Baseline pupillary measures (resting pupil diameter, parameters of pupil reflexes) should be presented in absolute units. It may be appropriate to use percentage changes in responses (e.g., after the application of an antagonist) only if the absolute sizes of the unaffected responses are available. Full details of the statistical analysis should be provided (e.g., for analysis of variance, F ratios and degrees of freedom, and not only levels of significance).

## 4. Psychology and Psychiatry Authors: Stuart R. Steinhauer and Kathryn A. Roecklein

### Introduction

Since the late 1950's, assessment of dynamic changes in the pupil (pupillography or pupillometry) have become a primary measure of increased cognitive and emotional activity (190–193). Both sympathetic and parasympathetic systems contribute to these pupillary modulations. The light reaction, which is primarily under parasympathetic control, can be reduced by emotional and cognitive activity. Suppression of the light reaction has been associated with fear and pain (194). Light can drive pupil constriction directly through the pupil light reflex, but also indirectly through retinal input to the suprachiasmatic nucleus and its pathways recruiting the dorsomedial hypothalamus and locus coeruleus, underlying wakefulness (193). Dilation in response to cognitive, effortful or emotional stimulation is mediated by both direct activation of the sympathetic system on dilator muscles of the iris, and by inhibition of the parasympathetic pathway leading to relaxation of the sphincter muscles (195–197). As in pure physiological experiments, the interaction of these systems may involve considerable reciprocal inhibition: the stimulation of one pathway is accompanied by decreased activity in the complementary pathway. The PIPR, described in Chapter 1, is a third type of pupil response potentially affected by psychological processes and is the dilation after illumination offset that persists as a function of melanopsin cell responses (8, 9).

### Stimulus Characteristics

The characteristics of stimuli that elicit pupillary dilation, or that modify parameters of the light reaction, are related to virtually all sensory modalities, and are sensitive to different contextual states. Thus, in relation to psychology and psychophysiology, there are three essential domains that need to be considered [after Sutton (198)]: (1) the physiological response (in this case, modulation of the pupil), (2) stimulus characteristics, and (3) the contingencies for behavioral response and task demand. In contrast to absolute stimulation and analysis approaches employed in clinical ophthalmological work, psychological and neuropsychiatric research employing pupillary assessment does not involve any standardized paradigms and is more often related to the parameters of complex instructions and varying complexity in stimuli. Reporting characteristics discussed and adopted at the 1999 meeting of the International Colloquium on the Pupil (ICP99) are provided below and serve as standards for reporting.

#### Stimuli

In most non-psychological research, the varying stimulus element is light. For psychological studies, there are also changes in auditory and even more rarely, tactile or olfactory stimuli (the latter not discussed further). Light stimuli have their most direct effect in producing constriction of the pupil, but in psychological studies, complex visual stimuli are often used to convey different meanings. Thus, the classical digit span task involves presenting a series of auditory stimuli which are later repeated, but the same effect psychologically could be produced by presenting brief visual digits. Specific experiments may provide a visual background after which target stimuli are presented. When the difference between background and stimulus is significant, a light reaction may be produced, which confounds the accurate assessment of dilation to the task demands in several potential ways: the light reaction may be magnitudes of order greater than the dilation, or baseline from which the dilation is measured may be shifted. At the very least, the luminance of the display should be specified in candelas/square meter (cd.m^−2^; ICP99). For single discrete stimuli, it is often possible to report the wavelength in nanometers. This is more difficult when using complex pictures, which vary in brightness across the visual field. One approach to minimizing hue effects (and stimulation of different photoreceptors) is to transform pictures into gray scales (199). For example, when presenting words, numbers, or small figures, use of black stimuli on a gray background minimizes contrast effects, and using pre- and post-stimulus masks (a row of X's, then the target, then X's again) also tends to minimize contrast effects (200). In such cases, the size of the stimuli needs to be provided in degrees of visual angle (which can be calculated using actual size and distance from the display). Distance of the visual stimulus from the eye is a consideration, as very close stimuli will result in constriction of the pupil related to vergence and accommodation effects.

Similarly, auditory stimuli need to be specified in loudness and duration. For pure tones, frequency should be specified, though this is not practical for spoken words or other complex sounds. Except for abrupt transients (that can elicit orienting reflexes), rise and fall times for auditory stimuli are not so critical in pupillary studies as in electrophysiology.

Finally, there are interactions related to the illumination of the testing situation. Pupillary oscillations are always greater in the presence of increasing ambient light, which decreases signal-to-noise ratio. Thus, recording in darkness minimizes oscillations, though provides more emphasis on sympathetic activation than parasympathetic inhibition. In darkness, there may be a ceiling effect on maximum pupillary dilation.

#### Behavior

This aspect is related to the task demands in psychological experiments. The participant may be asked just to sit passively, but most studies involve an interaction based on instructions. There may be cues that instruct subjects to have different expectancies (which increases pre-stimulus diameter), or to remember and modulate responses to stimuli (remember and repeat; calculate; sort numbers; categorize). The parameters of procedures and instructions to subjects are critical to communicate to readers. It is not unusual to ascribe a complex psychological context to a task manipulation, but without knowing exactly what the subject is being asked, it is difficult to know whether the proposed construct has actually been implemented.

Minor instructional differences can have significant effects. For example, asking a subject to make a simple button press every time a tone occurs seems overtly simple, and results in a dilation beginning around 500 ms and peaking around 1,200–1,400 ms. If instead the subject is asked just to make a voluntary press every few seconds, the early portion of the response is seen, but with a smaller dilation that ends before 1,000 ms. Even the presence of an experimenter near the participant can influence pupillary findings (201).

### Analysis

#### Initial Pupillary Recording and Data Reduction

Most current pupil and eye tracking devices have a minimum temporal resolution of 50 or 60 Hz, though some handheld devices use a slower sampling rate. As maximum frequency response of the pupil is <9 Hz, even a 20 Hz sampling rate is enough to capture critical aspects of pupillary oscillations. The pupil has a relatively large signal-to-noise ratio so that for most processing tasks, use of repeated conditions and averaging of the same condition provides a waveform that eliminates artifacts due to other factors, though only 5–10 repetitions of a condition may be necessary compared to the larger number of repetitions needed for event-related potentials and other physiological measures. In many studies, there may be up to 40 repetitions of a condition contributing to an average for an individual. Electrical noise, accuracy of edge detection of the pupil, and resolution of the recording device all may add some noise to the signal. The resolution of the recording system in mm should be specified—is it accurate to the nearest 0.02 or 0.05 mm or better? Some instruments provide a number that is confusing—the data file may give pupil diameter to the nearest 0.0001 mm, but this is not meaningful, it is a rounding error of the manufacturer. Most of the more accurate systems either provide direct measurement or a means for calibrating measurements to a known standard.

Preprocessing of the data to eliminate blinks or other artifacts is mandatory; short-duration artifacts can be corrected by linear interpolation between valid points (except at peaks and troughs of the signal). It is reasonable to filter pupillary data that have a high sampling rate (this can be easily performed by averaging of points around each original point, though peaks and troughs will be slightly attenuated). Filtering can be performed either before or after signal averaging. However, filtering and averaging can make determination of abrupt latency changes (time of light reaction or dark reaction onset) less precise.

#### The Parameters of the Light Reaction and PIPR

The parameters of the light reaction and PIPR are more clearly detailed in Chapter 1. For most psychological studies, the key measures will be prestimulus diameter (which can also be determined from onset of the light stimulus until the beginning of the light reaction), latency of the light reaction, and amplitude and latency of the light reaction. Other measures may include times to reach greatest constriction velocity, and times at which 50 or 75% of redilation are reached. Note that for very brief stimuli, there may be an incomplete light reaction (2), and for prolonged light stimuli, the pupil will begin to enlarge (pupillary escape) after the initial constriction.

#### Measures Related to Pupillary Dilation

Measures related to pupillary dilation are more complex and variable across studies as appropriate. The pupil may show a slowly increasing tonic change as working memory load is gradually increased, or phasic changes during the 1–2 s after presentation of more discrete stimuli. From the average response, it is somewhat standard to use a pre-stimulus average of 500–1,000 ms as a baseline diameter. Where a simple peak is observed, either amplitude of the peak (or simple average of a few points around the peak within a pre-specified range) may be calculated after subtracting the baseline diameter. In some experiments, the difference between baseline diameters by condition may be of interest. In experiments in which there is differential processing complexity, the peak may be delayed, as seen when sorting increasing numbers of digits (202) so that either amplitude or latency to peak may be of interest. In some experiments, including complex processing of emotional stimuli, there may be a prolonged dilation with no clear peak, resulting in a need for an average measurement over a prolonged interval rather than a specific peak. Measurement of dilation may be complicated by the occurrence of multiple peaks in the pupillary dilation waveform. When recordings are obtained in relatively bright conditions, there may be both an early dilation related to parasympathetic inhibition, as well as a later peak related to both parasympathetic inhibition and sympathetic activation.

#### Diameter/Change in mm vs. per Cent Change

The ICP99 standard was that pupil diameter in mm should be reported, rather than area or radius. There is also a question as to whether absolute measures of diameter or change in diameter should be reported, as compared to % change in baseline. Per cent change is often used in ophthalmologic practice to evaluate change across treatment conditions (e.g., % reduction of the light reaction due to pharmacological instillation). However, in psychological experiments, it is important to evaluate the actual change in diameter, since this may vary across conditions, as well as different baselines across conditions. Even where a rationale for using % change is presented, some reference to absolute pupil diameter and pupil change is necessary in order to replicate findings. Note that similar real changes in diameter will be underestimated for participants with larger initial diameter compared to smaller pupil diameter, a major problem in utilizing per cent change (203).

#### Statistical Analyses

Several alternative statistical approaches have been used. When comparing groups or variables within a group, *t*-tests or ANOVA models to evaluate differences in maximum constriction, peak dilation, or latency are most often appropriate. Often, there is interest in evaluating prolonged periods of pupillary activity rather than peak measures. A Principal Components Analysis (PCA) can be used to isolate orthogonally independent factors, which describe for each component how it is related to variation over the time of the waveform; factor scores may be derived which can then be subject to separate analyses (204). Another approach based on the Guthrie-Buchwald (205) procedure provides a minimum number of successive points that must all be significantly different across conditions (by *t*-test or ANOVA) to define *post-hoc* regions of significant effects (200). There has recently been increased interest in utilizing Bayesian statistics to define regions of significant differences.

#### Abbreviations

There is often much confusion in the pupillary literature associated with unique or uninformative abbreviations. Acceptable and readily recognized abbreviations include PLR (pupillary light reaction) or PIPR (post-illumination pupil response). All other abbreviations are recommended to be mnemonic as appropriate to a paper: for example PkDil or AvgDil communicates peak or average dilation (which still needs to be defined as the absolute value or difference from baseline). Beatty (206) had earlier introduced TEPR (task evoked pupillary response), but this is a confusing terminology as it referred to dilations, but could as easily be misinterpreted as a constriction response. Even PDR for pupillary dilation response is confusing and probably should be avoided.

### Application

Loewenfeld documented the two millennia history of pupillary movements in her dissertation (207) and epic tome on the pupil (2). The more sustained interest in psychological constructs was initiated after 1960 with studies of dilation in response to emotional stimuli (208) working memory tasks (209), orienting stimuli (210), and processing load (206), among others. Responses to orienting or novelty, emotionally salient, and simple feedback stimuli tend to peak at slightly after one second (193, 211–213). Mental effort or arousal responses which require greater processing time occur with greater peak latencies related to the complexity of the task, such as performing arithmetic (214), memorizing digits using working memory (202, 209), both positive and negatively salient arousal (199), reward processing (215), as well as numerous other types of cognitive or emotional processes (193). Convergent validity for pupillographic measures of arousal comes from correlation with psychophysiological measures of arousal such as skin conductance [e.g., (199)], and associations with pupillary and electrocortical activity [e.g., (211)]. Change in pupil diameter with alertness and sleepiness, including decreased diameter and increasing pupillary oscillations, are discussed in Chapter 5. Thus, pupillary assessment continues to be employed as a significant window on complex psychological processes (193). The pupil has been a strong investigative tool in psychopathology for over 70 years (particular schizophrenia and depression studies), with well documented decreased processing-related dilations in schizophrenia (204, 216, 217) and enhanced dilations to negatively valenced stimuli in depressed patients (200, 218, 219).

More recently, the PIPR has been evaluated among individuals with seasonal affective disorder (SAD), and is highlighted here in somewhat more detail. Initially, Roecklein et al. (41) found a reduced PIPR in SAD participants compared to nondepressed, nonseasonal participants. Laurenzo et al. (220) subsequently reported an attenuation of the PIPR in those with nonseasonal Major Depressive Disorder (MDD) compared to nondepressed controls, but only when using low intensity red and blue stimuli, and not under higher intensity chromatic stimuli. A seasonal variation was identified such that high intensity blue light responses in the post-illumination period were more pronounced during longer photoperiods (220). This is in contrast to the findings of Roecklein et al. (under review) presented in this issue. In a study of Age-Related Macular Degeneration (AMD) and healthy controls, AMD was associated with depression, but the PIPR was not correlated with depression (30). Depression was measured with a short self-report questionnaire and mean scores for both groups were below the cutoff of 16 indicating that the AMD group was, on average, not reporting symptoms of clinical depression. All individuals in this study were below self-report threshold on a screening implement for seasonal affective disorder. Münch et al. (221) reported a larger PIPR in winter in individuals without cataracts, and low levels of depression which is consistent with the seasonal variation found by Roecklein et al. (under review) in the present issue. Discrepancies, while few, in the emerging literature attempting to evaluate melanopsin cell responses to light in seasonal and nonseasonal depression have largely motivated the above review of melanopsin oriented pupillometry methods. Because light can impact both mood and learning and memory processes through melanopsin pathways (222), future work may employ the PIPR in studies on learning and memory as well as depression.

## 5. Sleepiness-Related Pupillary Oscillations Authors: Barbara J. Wilhelm, Kathryn A. Roecklein and Tobias Peters

### Introduction

Oscillations of pupil diameter in darkness related to sleepiness of a subject were first described by Lowenstein et al. (223) and called “fatigue waves” at that time. Today, there is a differentiation between sleepiness (related to either quantity or quality of sleep) and fatigue (not necessarily related to sleep, but also evocable by physical or psychological exhaustion) in sleep research and sleep medicine. Therefore, the terms “sleepiness waves” and “sleepiness-related oscillations” are preferable because fatigue does not result in oscillations in pupil diameter. Lowenstein et al. (223) confirmed the central nervous system origin of sleepiness-related oscillations of pupil diameter in darkness using pharmacological experiments. Subsequently, Yoss et al. used the Lowenstein device clinically in the diagnosis and treatment of patients with narcolepsy and developed a classification of pupillary oscillations related to eye lid movements and EEG signs of increased sleepiness (224, 225). In the following two decades few clinical applications emerged due to complicated apparatuses and a lack of automation (226, 227). The pupillographic sleepiness test (PST) developed by Lüdtke et al. (228) utilized modern technical possibilities regarding recording, image analysis, artifact elimination and automated analysis. Classical test quality criteria of the PST have been evaluated and are adequate (229–231). The PST is now established and widely used in sleep research and sleep medicine as well as in psychology (232–234).

### Experimental Conditions for the Pupillographic Sleepiness Test

#### Darkness

Light is the major contributing factor of the pupil diameter. To capture measures of autonomic arousal, all light sources need to be excluded (235). Therefore, infrared goggles are used for the recording of sleepiness-related pupillary oscillations. Depending on individual face shape such goggles may not be completely light-tight and for this reason the examination room should be as dark as possible (in the mesopic range, i.e., below 3 cd.m^−2^) and the illumination level needs to be quantified. Infrared indicators can be used for orientation.

#### Silence

It is important to protect the subject from acoustic influences in a silent room with sound dampening or by the use of noise canceling headphones. During the recording period communication with the participant is prohibited and the examiner is meant to be silent during examination.

#### Temperature

Room temperature needs to be comfortable because cold temperatures stimulate the sympathetic nervous system and may have an alerting effect. In addition, high temperatures may result in higher pupillary unrest index (PUI) due to reduced alertness. Stable room temperature between 68 and 72°F (20–23°C) is ideal.

#### Time of Day

Normative values for the PST have been collected during the first half of the day [8 a.m. to 1 p.m.; (231)]. Patients with obstructive sleep apnea show their highest sleepiness values during this timeframe. For hospital use, this time frame is recommended to allow for comparison with the reference values.

#### Medication

Topical medication (eye drops) with effects on pupil size should be avoided prior to testing. In addition, systemic medications with psychoactive or alerting effects or influence on the sympathetic/parasympathetic nervous system should be avoided if feasible or documented. The PST can also be used to quantify improvement in sleepiness due to a therapeutic regimen when measured pre-treatment and post-treatment.

#### Caffeine and Nicotine Consumption

Participants should be reminded to abstain from caffeine for 8–10 h prior to testing (236). However, this recommendation may be relaxed in field studies to avoid withdrawal effects or poor compliance. Nicotine has a minimal impact on the PUI and performing the PST 1 h after the last use of tobacco or nicotine products is sufficient (237).

#### Preparation and Standardized Instruction of the Participant

Reference values have been collected previously only after a 10 min period of sedentary rest to minimize any impact of physical activity on the PST (238). The following verbal instructions have been provided in past studies, including those reporting normative PUI values, and are provided here to be used broadly in future studies as a standardized set of instructions prior to recording the PST.

“The measurement will last 11 min. During the recording it will be dark and quiet in the room. We will not talk to you before the recording is completed. Please look in the direction of the red fixation light; you do not need to focus on it.

You may want to avoid thinking about problems or plans for your day, or about difficult issues in your life. Just look straight ahead and relax. We will now adjust the camera and we will inform you when the recording is about to start.”

#### Preliminary Pupil Examination

Before the pupillographic sleepiness test is started, a basic pupil examination (160) should be performed with a flashlight or other light source to make sure that the recorded pupil shows normal and unrestricted mobility.

#### Test Duration and Interval of Consecutive Measurements

The standard test duration, which also has been used when collecting normative values, is 11 min of recording (230, 233). In the case of series of recordings an interval of 2 h is recommended to avoid possible sequence effects.

#### Data Surveillance During the Recording

Because participants may fall asleep, or the camera may need to be adjusted if data loss occurs, experimenters should monitor data acquisition continuously either in the same room or from outside of the testing room via monitor to ensure the quality of the recording.

#### Falling Asleep

If a participant falls asleep during testing, we recommend that the experimenter provides a brief acoustic signal if the participant does not awaken in 20 s. A louder or longer signal should be used if the first is unsuccessful, and a verbal request to open the eyes may be delivered by the examiner, if ultimately necessary. We recommend that all such sleep events should be documented, and that a test with multiple sleep events should be considered as pathological and/or as excessive sleepiness.

#### Subjective Rating of Sleepiness

The Stanford Sleepiness Scale or the Karolinska Sleepiness Scale assess self-reported sleepiness and can be included to determine the degree of awareness or insight into the degree of sleepiness retrospectively after recording in the clinical setting.

#### The Suggested Experimental Order of Procedures Is:

1. 10 min of physical rest
2. Preliminary pupil examination
3. Assessment of medication, caffeine, and nicotine use; entry into database
4. PST recording for 11 min
5. Subjective sleepiness scale.

### Analysis

Before analysis of a recording, high frequency noise (e.g., due to blinks) is excluded and, for missing values, a linear interpolation is applied which is standard in pupillographic recordings in general (239). Parameters of evaluation may be gained by Fast Fourier Transformation (FFT) or calculation of the Pupillary Unrest Index (PUI). FFT normally is characterized by the amplitude spectrum (or “power”) in the frequency range ≤0.8 Hz (228). The PUI is the sum of the absolute changes in pupil diameter over the time of recording and is given in mm/min (230). For statistical analysis the natural logarithm of the PUI is recommended due to its normal distribution in larger samples (231).

### Data Reporting Recommendations

- Participants
  Besides general aspects as age and sex (see general standards Part I), the time lag to the consumption of nicotine and caffeine should be provided.
  In addition: sleep habits, method of assessing sleep behavior during the days before the recording (e.g., diary, actigraphy), use of alerting or sedative medication.
- Technical information on test system, camera (video frequency in hertz), sampling rate, image analysis.
- Method of artifact management. Specify methods for blink removal.
- General conditions of the pupillographic sleepiness
  Information on all conditions listed above should be provided. Deviations from these standard conditions should be described and substantiated.
- Pupil parameters (averages of the recording period): pupil size (absolute), Pupillary Unrest Index (PUI, absolute and ln) and interpolation rate should be given for the investigated sample.
- Classification of test result in normal, suspicious or pathological, according to the “green (±1 SD), yellow (between 1 and 2 SD) or red (above 2 SD)” flag in relation to normative sample (231).
- Number of recordings with sleep events, if occurred. Such recordings should be classified as pathological, regardless of PUI value.

## 6. Animals Author: Paul D. Gamlin

### Introduction

Pupillary responses in animals, as in humans, are driven by the parasympathetic and sympathetic components of the autonomic nervous system (131), and are studied for a number of reasons. First, animals serve as potential models for humans for understanding the retinal and central processing of pupillary control signals, both those driven by light and those modulated by eye movements, attention, or cognition. Second, pupillary responses can be used to assess animal models of retinal degeneration. Third, pupillary responses can be used to assess autonomic function. Fourth, the discovery of intrinsically photosensitive retinal ganglion cells (ipRGCs) and their contribution to a non-image forming visual system that drives pupillary responses, entrains circadian rhythms, and can affect sleep and mood has resulted in the pupil being studied as a surrogate for, or to complement, studies of these other systems. Indeed, all mammals studied to date show pupillary responses consistent with rod, cone, and melanopsin driven responses [e.g., (6, 8, 13, 96, 97, 240)].

In this chapter, we propose some standards that should be followed when studying pupillary responses in animals.

### Stimulus Characteristics

In studies of light-evoked pupillary responses, it is important to fully characterize the visual stimulus. Authors should specify: (1) the spectral content or the light source, and provide either the corneal or estimated retinal irradiance (121); (2) the spatial extent and retinal locus of the stimulus; (3) the duration of the stimulus; (4) the duration of recording–ensuring that a period of time prior to the stimulus is recorded for baseline purposes; (5) whether the stimulus is binocular or monocular; (6)whether either pupil is dilated pharmacologically; (7) whether pupil responses are measured monocularly or binocularly; (8) whether direct or consensual pupil responses were measured.

### Restraint

In many cases, animals will be restrained by either physical or chemical means. Each approach presents a challenge to reliable measurements of the pupil. In the case of physical restraint, whether it consists of body and head restraint, or just head restraint, animals must undergo significant acclimation, usually with positive reward, such that their stress responses are minimal when pupillary measurements are taken. Failure to do so will result in increased sympathetic tone, and unreliable measurement of resting state pupil diameters and light-evoked responses. In case of chemical restraint, the challenges are greater, and the ability to compare results between laboratories will rely heavily on the use of the same chemical restraint protocol. Further, the results obtained under chemical restraint are unlikely to match those that would be obtained from a physically restrained, but relaxed animal.

### Species Specific Recommendations

#### Monkeys

##### Sedated Protocol

In some cases, pupillary responses can be studied in sedated monkeys. Animals in such studies will not require the surgically implanted head holder that is generally used for studies in alert monkeys. For this procedure, one acceptable protocol is as follows: animals are lightly anesthetized (heart rate maintained at awake levels) using intramuscular injections of a low dose of anesthetic (<10 mg/kg ketamine and 0.1 mg/kg acepromazine) with supplementation as needed to maintain anesthesia. If the heart rate is seen to decrease, this is a sign that the level of anesthesia is too deep and will result in suppressed pupillary responses. The head of the animal is stabilized by a bite bar and head holder.

##### Alert Protocol

In almost all cases, monkeys will be chair-trained, will receive a surgically implanted head holder, and will be extensively acclimated to head fixation for periods of up to a few hours while viewing visual targets for liquid or food reward. Such procedures have been used in studies of the pupillary light reflex in macaques [e.g., (8)] and cognition [reviewed by Binda and Gamlin (241)].

For open-loop studies of the pupillary light reflex, either the stimuli should be presented to one eye in which the pupil has been dilated, or the stimulus duration should be brief enough to ensure that it is extinguished prior to pupil constriction. For closed-loop experiments, in which pupil constriction alters retinal irradiance, no such limitations are necessary. In general, pupil diameters should be measured in both eyes under infrared illumination using video cameras. For open-loop experiments, the pupil of one eye will be dilated with 1.0% tropicamide and 2.5% phenylephrine. Therefore, pupillary responses elicited by stimuli presented to this eye are measured by evaluating the consensual pupil response of the fellow eye. Animals fixate with the fellow eye on a target presented on a computer monitor. Pupil diameter in the fellow eye is monitored continuously. After a period of fixation, a stimulus is presented to the eye with the dilated pupil. The duration, extent, intensity, and spectral content of the stimulus will be appropriately varied for the planned experiment. The stimulus is then extinguished, and the participant maintains fixation for up to 30 s (8).

For studies of cognitively-related pupil responses, very similar procedures to the above are followed. The animal is head-fixed performing a behavioral task for water or food. In general, pupil diameters are measured by the eye movement systems used in these experiments. If the pupil is to be measured at anything other than primary position, then the investigator should try to calibrate pupil measurements throughout the range of expected eye movements, and should not rely solely on the cosine correction factors that are often used by these systems.

For these alert monkey protocols to yield reliable data, it is essential that the animal is fully acclimated to both the required head fixation and the task, and be actively engaged in the required task. Pupillary hippus and signs of sleepiness should be monitored since, as in humans, these will affect resting pupil diameter and light-evoked pupillary responses.

#### Dogs

While it is feasible to measure resting pupil diameter in conscious dogs (96), studies of light-evoked pupillary responses generally use chemical restraint. In an early study, dogs were mildly sedated with medetomidine administered intramuscularly in a dose of 5 micrograms/kg body weight (240). In later studies, this level of sedation was found to be unsuitable for more extensive pupil light reflex testing. Whiting and colleagues (96) evaluated five different chemical restraint protocols for measurement of the pupillary light reflex in purpose–bred long-haired miniature Dachshunds. They found that 5 μg/kg dexmedetomidine provided them with insufficient restraint to place a speculum, while higher doses of dexmedetomidine alone (35 μg/kg) or dexmedetomidine/ketamine (18 μg/kg/3.5 mg/kg) or dexmedetomidine/butorphanol (5 μg/kg/0.17 mg/kg) resulted in large spontaneous fluctuations in pupil size. Therefore, they recommended the following protocol: Dexmedetomidine (20–25 lg/kg IM) is given 30 min before induction of anesthesia with propofol (intravenous [IV] to effect, 1.49 ± 0.59 mg/kg [mean ± SD]). Dogs are intubated with a cuffed endotracheal tube and restraint maintained with 1.5% isoflurane in oxygen. This protocol is similar to that used recently by Yeh and colleagues in both wild type dogs, and dogs with retinal and optic nerve disease (97). In this study, animals were first premedicated using acepromazine, at an intravenous dose of 0.02 mg/kg and induced with propofol given intravenously to effect (starting dose, 4 mg/kg). The dogs were then intubated, and general anesthesia was maintained with 2–3% isoflurane in oxygen. The differences between the two studies in the propofol and isoflurane doses used may result from the different breeds of dog used in these studies.

#### Mice

PLR testing can be conducted in completely awake mice without the use of general anesthetic or sedation. The mice are initially habituated to extensive handling with food rewards, in order for them to remain calm during recording [e.g., (242)]. Acclimated animals can be lightly held by the scruff of the neck to ensure they are correctly positioned during pupil measurements without increased stress (S. Hattar, Personal communication). In most studies, the steady state pupil diameter (~30 s light) is measured. Prior to stimulation, animals are usually dark adapted for 1 h. For ease of measurement, one eye is usually exposed to the stimulus while the consensual pupil response is measured. Animals can also be acclimated to head fixation [e.g., (243, 244)], and similar procedures followed for measurement of the pupillary light reflex.

## Final Remarks

The authors understand these standards in pupillography as a “living” standard; new insights in retinal and pupillary circuitries as well as in stimulation and response analysis parameters of future projects will consequently be amended.

## Author Contributions

The introduction and I. Part were written by CK and TS; II. Part: 1. CK, AZ, BF, HW, YC, PG, RK; 2. HW; 3. ES; 4. SS, KR; 5. BW, KR, TP; 6. PG. Additional details are included within the manuscript sub-parts. All authors critically reviewed the manuscript.

### Conflict of Interest Statement

The authors declare that the research was conducted in the absence of any commercial or financial relationships that could be construed as a potential conflict of interest.

## Footnotes

^1^https://iscev.wildapricot.org/
