# Erratum: Standards in Pupillography

**DOI:** 10.3389/fneur.2019.00371

**Published:** 2019-03-27

**Authors:** Frontiers Production Office

**Affiliations:** Frontiers Production Office, Frontiers Media SA, Lausanne, Switzerland

**Keywords:** clinical standards, pupillography, application of pupillography, stimulus characteristics, parameters of evaluation, analysis, pupillometry

Due to a production error, the funder “Egon Schumacher-Stiftung, Barnstorf, Germany”, a private foundation without commercial interest (CK, TS, TP, BW, HW), was incorrectly omitted from the funding statement.

The publisher apologizes for this mistake. The original article has been updated.

